# The perfect storm: Thrombotic thrombocytopenic purpura (TTP) associated with COVID‐19, a clinical case series and review

**DOI:** 10.1002/jha2.577

**Published:** 2022-10-26

**Authors:** Neumann Melissa, Singhal Adit, Habibullah Junaid, Dhar Sean

**Affiliations:** ^1^ Donald and Barbara Zucker School of Medicine at Hofstra/Northwell Manhasset New York USA; ^2^ Division of Hospital Medicine and Division of Pulmonary Critical Care and Sleep Medicine Donald and Barbara Zucker School of Medicine at Hofstra/Northwell Manhasset New York USA

**Keywords:** ADAMTS, COVID‐19, PLASMIC, thrombotic microangiopathy, thrombotic thrombocytopenic purpura

## Abstract

This is a case series of three patients in our hospital system who developed acquired thrombotic thrombocytopenic purpura (aTTP) after testing positive for COVID‐19 infection. Two patients had acute COVID‐19 infections, and one had COVID‐19 IgG antibodies consistent with prior COVID‐19 infection. Twelve additional cases of aTTP after COVID‐19 infection were found in the literature. COVID‐19 creates alterations in the vWF‐ADAMTS‐13 axis with reduced ADAMTS‐13 in acute illness that may lead those patients who are predisposed into fulminant aTTP. Further genetic studies are necessary to uncover why some patients with COVID‐19 can have concurrent aTTP. For those with a prior COVID‐19 infection, molecular mimicry with autoantibodies to ADAMTS‐13 is likely the predominant trigger, but having an underlying predisposition (prior episode of TTP, genetic predisposition to autoimmune conditions, or breast cancer history) creates an environment that could be a possible trigger for aTTP.

## 1 INTRODUCTION

This is a case series of acquired thrombotic thrombocytopenic purpura (aTTP) associated with the novel SARS‐CoV‐2 virus (COVID‐19). COVID‐19 has been linked to numerous different autoimmune diseases with a likely mechanism being the immune response to a self‐antigen that is structurally similar to a viral protein, called molecular mimicry [1, 2]. In this report, we describe three new cases of COVID‐19‐induced aTTP in addition to 12 cases found in our literature review. We found two subsets of patients, those with an acute COVID‐19 infection (a positive nasal polymerase chain reaction [PCR] for COVID‐19) and those with a prior COVID‐19 infection (negative PCR but positive COVID‐19 IgG antibodies). Likely molecular mimicry plays a role in both forms of aTTP in patients with predisposing factors. ADAMTS‐13 dysfunction is the main driver of aTTP, and the antigen can be measured by enzyme‐linked immunosorbent assay, which are time consuming (from 2 to 4 days) with various substrates (plasma‐derived, recombinant, and flurouescence energy transfer substrates). There are numerous limitations in fluorescent assays include interferences from plasma proteins such as bilirubin and hemoglobin impeding accurate measurements at low ADAMTS‐13 activity. Nonetheless, an ADAMTS‐13 activity level of <5%–10% is specific for aTTP and increases in specificity if an inhibitor is present [3, 4, 5]. Evaluating levels of von Willebrand Factor Antigen (vWF:Ag) may be warranted for COVID‐19‐induced TTP, since this correlates with disease severity. Understanding these mechanisms and identifying patients at hospital presentation through calculating PLASMIC scores are important for early treatment of this deadly disease.

### 1.1 Case presentation

This is a case series of aTTP due to the novel SARS‐CoV‐2 virus (COVID‐19). We present three patients.

**GRAPH 1:**
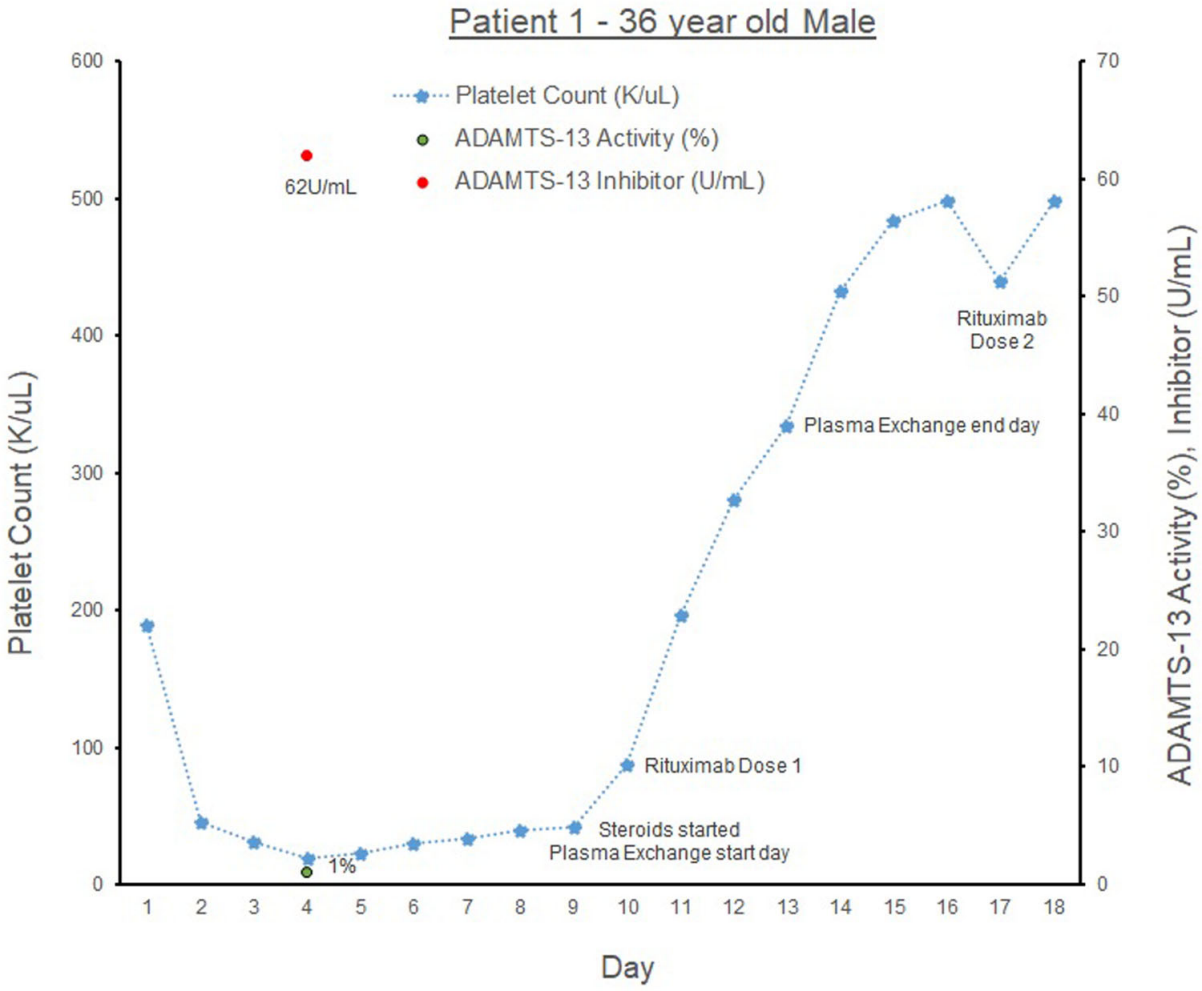


Our first patient (see graph 1) is a 36‐year‐old male with hypertension, alpha‐thalassemia trait, who presented with abdominal pain. Family history includes a sister with idiopathic thrombocytopenic purpura (ITP). The patient was found to have cholangitis and was treated with antibiotics and an endoscopic retrograde cholangiopancreatography with biliary stent placement.

At admission, his initial laboratories showed hemoglobin 11.2 g/dl (normal *n* = 13–17 g/dl) and platelet count (PLT) of 209 K/μl (*n* = 150‐400 K/μl), and total bilirubin (Tbili) 4.8 mg/dl (*n* = 0.2–1.2 mg/dl). On COVID‐19 screening, he had COVID‐19 IgG antibodies but was negative on COVID PCR testing. On day 2 of admission, the patient's PLTs acutely dropped to 45 K/μl, and his Tbili increased to 20.8 mg/dl, with direct bilirubin (Dbili) 14.1 mg/dl (*n* = 0.0–0.2 mg/dl). Laboratories were notable for lactate dehydrogenase 908 U/L (*n* = 50–242 U/L) and haptoglobin <20 g/L (*n* = 34–200 g/L). His PLTs dropped further to 19 K/μl. His ADAMTS‐13 Ab (ADAMTS‐Ab) was found to be elevated at 62 U/ml (*n* < 12) and activity level (ADAMTS‐AL) <2% (*n* > 66.8%), consistent with aTTP. Peripheral smear revealed schistocytes. The patient was treated with plasma exchange therapy (PLEX) for 5 days and 1 gm of Solumedrol once a day for 3 days followed by a prednisone taper. He was discharged 10 days later with PLT of 498 K/μl and weekly Rituximab 874 mg IV for 4 weeks. He has had no further relapses.

**GRAPH 2:**
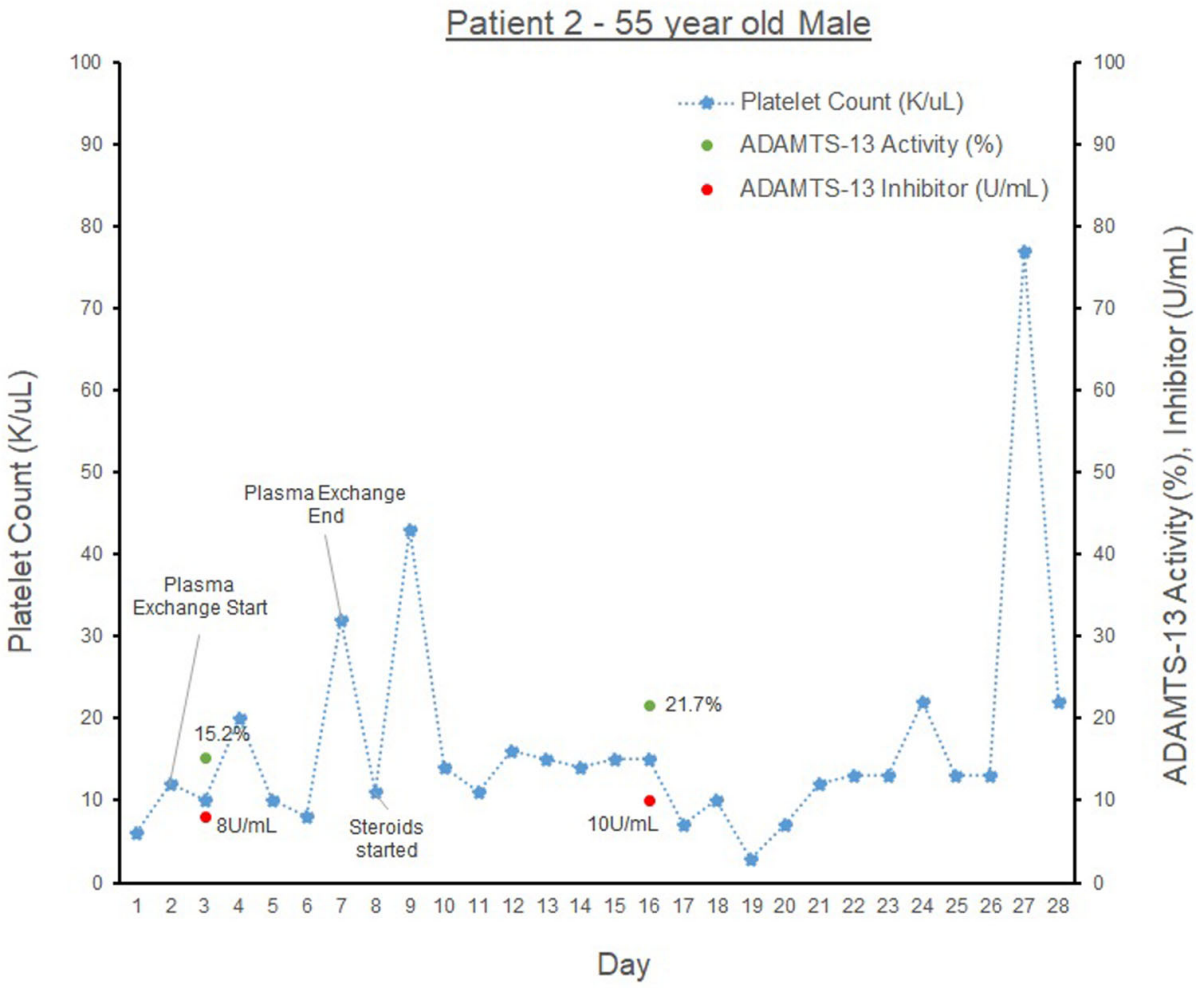


Our second patient (see graph 2) is a 57‐year‐old male with congestive heart failure, hypertension, coronary artery disease presenting with shortness of breath and thrombocytopenia. On physical exam, the patient was hypoxemic with an oxygen saturation of 70% and jaundiced. Laboratories were significant for potassium 6.4 mmol/L (*n* = 3.5–4.3 mmol/L), sodium 114 mmol/L (*n* = 135–145 mmol/L), creatinine 3.5 mg/dl (0.5–1.3 mg/dl), and hemoglobin was 6.0 g/dl and PLT 8 K/μl, Tbili 13.1 mg/dl, Dbili 12.1 mg/dl, haptoglobin <20 g/L, C‐reactive protein 250.6 mg/L (*n* < 4.9 mg/L), ferritin 14,428 ng/ml (*n* = 30–400 ng/ml), procalcitonin 48.5 ng/ml (0.02–0.1 ng/ml), pH 7.21 (*n* = 7.32–7.43), and bicarbonate 11 mmol/L (*n* = 22–31 mmol/L). He was diagnosed with COVID‐19 pneumonia, which progressed to acute respiratory distress syndrome complicated by aTTP. His ADAMTS‐AL was 15.2% and ADAMTS‐Ab 8 Units/ml. He had slight schistocytes on peripheral smear. He was admitted to the medicine intensive care unit (MICU) and treated with dexamethasone and remdesivir. He received nine units of packed red blood cells (pRBCs), four units of fresh frozen plasma (FFP), 10 units of cryoprecipitate, 17 units of platelets, and PLEX six times with steroids followed by IVIG. A bone marrow biopsy was performed, and his hospital course was complicated by suspected hemophagocytic lymphohistiocytosis, Klebsiella bacteremia, fungemia, endocarditis, and renal and multiorgan failure. He did not improve after PLEX and expired 1‐month after admission.

**GRAPH 3:**
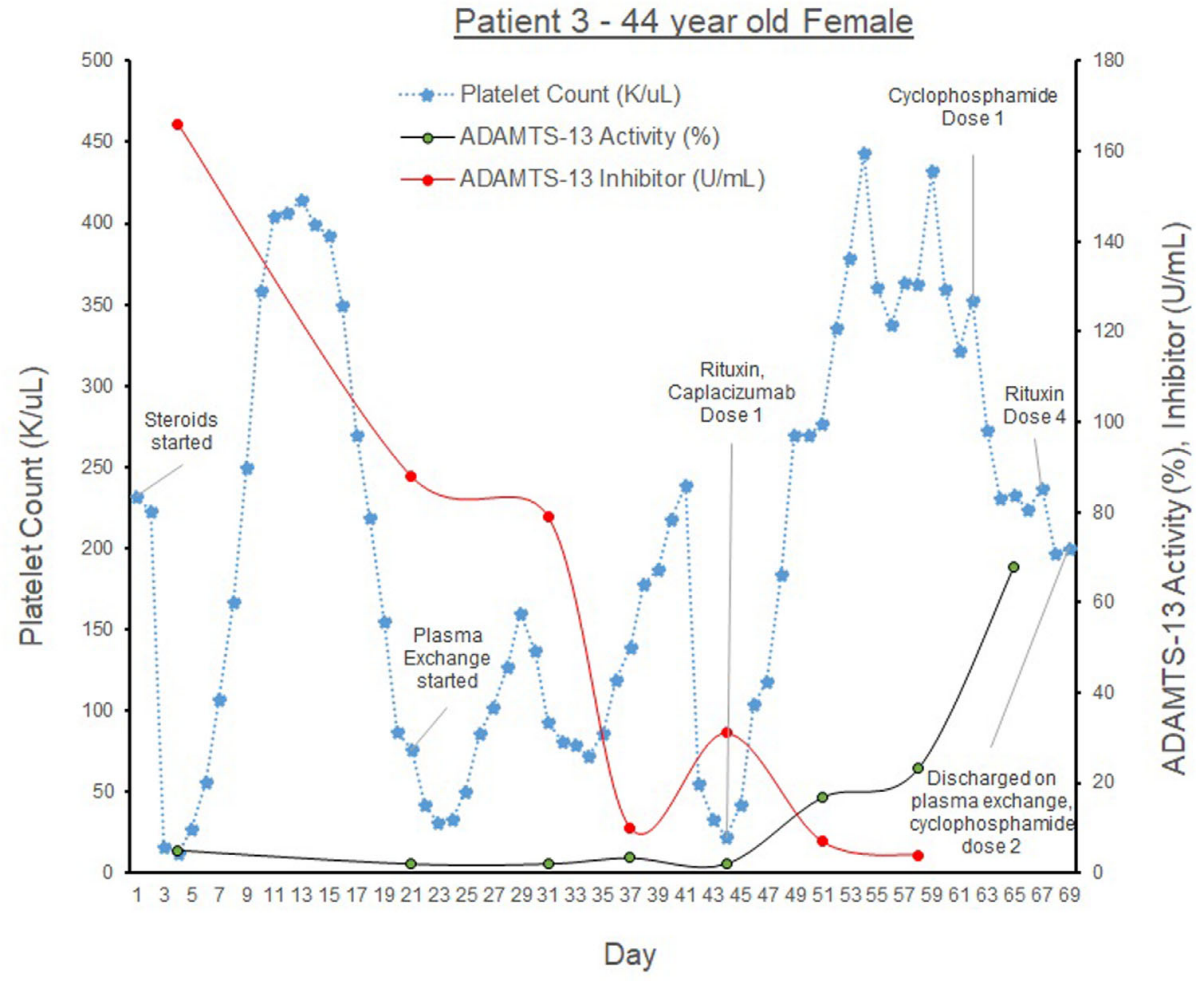


The third patient (see graph 3) is a 44‐year‐old female with hypothyroidism presenting with fatigue, weakness, and productive cough, found to have COVID‐19. On admission, her hemoglobin was 6.1 g/dl, platelets 10 K/μl, haptoglobin < 20 mg/dl, creatinine 10.8 mg/dl. Schistocytes were seen on peripheral smear, and ADAMTS‐AL was <2.0% with ADAMTS‐Ab 166 U/ml. She developed acute renal failure requiring hemodialysis and first‐time seizures. She received transfusions of pRBCs, platelets and FFP, steroids, PLEX x 6, caplacizumab 11 mg daily and was discharged with normal PLTs to subacute rehabilitation but was re‐admitted for TTP‐relapse after 2 days with PLTs 31 K/μl. She was transferred to the MICU for hemodialysis and treated with PLEX (until her [PLT] normalized), steroids, cyclophosphamide 600 mg /m^2^ twice, and rituximab once she was 1‐month out from her acute COVID‐19 infection. She was discharged with a steroid taper. A week later, although her platelets were 242 K/μl, she was re‐admitted for persistent COVID‐19 infection after receiving PLEX outpatient once. Inpatient, she received cyclophosphamide 600 mg/m^2^, and PLEX and was discharged with monthly cyclophosphamide and subsequent PLEX treatments. As far as we know, she has remained in remission and has had no further relapses.

## 2 DISCUSSION

TTP is a life‐threatening thrombotic microangiopathy (TMA) caused by increased platelet adhesion and aggregation. ADAMTS13 is a proteinase enzyme that cleaves ultra‐large Von Willebrand factor (ULVWF). When activity of ADAMTS13 is low, ULVWF is not cleaved, leading to ULVWF‐platelet aggregation and consumption of platelets. TTP diagnosis includes an ADAMTS‐Ab >15 U/ml and an ADAMTS‐AL <10%. However, ADAMTS‐AL between 10% and 20% may support the diagnosis of TTP in some individuals. The pathogenesis of TTP has been well studied and includes inherited TTP, driven by a gene mutation causing a deficiency in ADAMTS13 production and acquired TTP, which involves an autoantibody, and a decrease in ADAMTS13‐AL, which occurs in sepsis, liver disease, pancreatitis, and cardiac disease. Molecular mimicry by infectious diseases, such as viral infections, has long been identified as a trigger for auto‐immune disease. Molecular mimicry occurs due to the structural similarity between a foreign antigen and self‐antigen.

In this report, we describe three new cases of COVID‐19‐induced aTTP in addition to 12 cases found in our literature review [6, 7, 8, 9, 10, 11, 12, 13, 14] (Table 1: 3–11). All cases except three presented with an acute COVID‐19 infection; those with prior COVID‐19 infection included ours, Albiol et al. and Capecchi et al. All patients were treated with PLEX except one, a 57‐year‐old female reported by Tehrani et al. who was treated with FFP and IVIG due to a plasmapheresis shortage but had a positive outcome nonetheless. All patients were discharged except two, first being our 57‐year‐old male and second being a 56‐year‐old female who died due to hemorrhagic stroke [12]. Three cases did not report ADAMTS‐AL or inhibitor levels [8, 9, 10]. All other cases met the criteria for TTP with ADAMTS‐Ab > 15U/ml and ADAMTS‐Activity <10% with the exception of our 57‐year‐old male, which had borderline ADAMTS‐AL of 15.2%. A PLASMIC score, which predicts ADAMTS‐13 deficiency in suspected TTP patients, was calculated for the other patients when available (see Table 1). A PLASMIC score is used to assess the likelihood of a TMA being due to severe ADAMTS‐13 deficiency. This is based on variables such as PLT, hemolysis, active cancer, etc. [15]. In our study, PLASMIC scores correlated well and can be of utility when screening for COVID‐aTTP.

**TABLE 1:** Clinical characteristics and findings of case series and previously published reports of acquired thrombotic thrombocytopenic purpura (aTTP) and COVID‐19. Pentad refers to the presence of the five classical clinical findings of microangiopathic hemolytic anemia (MAHA), fever, renal dysfunction, anemia, and neurological symptoms. PLASMIC scores were calculated when possible, or marked with a “+” if not all components to calculate a score were available

																ADAMTS‐13		
	Age Gender	PMHx	Presenting Sx	Intubated	Time from Sx onset to thrombocytopenia	Pentad	WBC (K/μl)	Hgb (g/dl)	PLT (K/μl)	LDH (U/L)	SCr (mg/dl)	Haptoglobin (g/L)	Tbili (mg/dl)	Ibili (mg/dl)	Diff.	Activity	Inhibitor	PLASMIC Score
Neumann et al.	36 M	HTN, Alpha‐thalassemia trait	Abdominal pain	No	4 days (IgG only)	MAHA, thrombocytopenia	8.29	7.8	45	908	1.15	<0.2	20.8	6.7	Schistocytes	<2%	62	6
	57 M	CHF, MI, HTN	SOB	Yes	11 days	MAHA, fever, thrombocytopenia, kidney injury	21.02	5.4	6	1696	3.5	<0.2	13.1	1	Schistocytes	15.20%	8	6
	44F	Hypothyroidism	Fatigue	No	4 days	MAHA, thrombocytopenia, kidney injury	8.86	6	16	3415	8.23	<0.2	1.2	0.85	Schistocytes	<2%	166 U/ml	7
Albiol et al. [3]	57F	HTN, breast cancer (complete remission)	Dry cough, anosmia, dysgeusia	No	6 days (IgG only)	MAHA, thrombocytopenia	9	6.9	13	1594	0.72	–	–	0.86	Schistocytes	2%	5.2	5+
Capecchi et al. [4]	55F	TTP 2/2 bacterial pneumonia	Malaise, fatigue, chest discomfort, dyspnea	No	13 days (IgG only)	MAHA, thrombocytopenia, neurological dysfunction, renal failure	40.55	7.4	14	####	3.13	<0.1	9.22	2.12	–	Undetectable	31 U/ml	4+
Altowyan et al. [5]	39 M	Diabetes type II, hypertension	Epigastric pain, nausea, vomiting	No	–	MAHA, thrombocytopenia	14.6	6.7	6	1600	0.77	<0.2	4.8	2.7	Schistocytes	N/A	N/A	6
Al‐Ansari et al. [6]	51 M	Kidney transplant on immunosuppression, HTN, HLD	Fever, SOB, sore throat, nausea, body aches.	Yes	2 days	MAHA, fever, thrombocytopenia, kidney injury, neurological symptoms	8.3	6	20	757	2.27	<0.295	–	–	Schistocytes	N/A	N/A	N/A
Aminimoghaddam et al. [7]	21F	Pregnant (3rd trimester)	Fever, dry cough	No	7 days	MAHA, fever	11	5	21	1910	5.6	N/A	1.8	–	Schistocytes	N/A	N/A	4+
Beaulieu et al. [8]	70 M	PAD, HLD	Confusion	Yes	19 days	MAHA, thrombocytopenia, neurological dysfunction	8	6	18	1422	1.2	<0.3	0.43	–	Schistocytes	<10%	Weakly	6
Tehrani et al. [9]	25F	Pregnant (3rd trimester)	Severe respiratory symptoms	No	–	MAHA, thrombocytopenia, fever	22	7	10.5	3465	–	–	4	3.6	Schistocytes	8%	85	N/A
	56F	Breast cancer (remission)	Fever, SOB	–	–	MAHA, thrombocytopenia	14.6	6	41	1520	–	–	–	–	Schistocytes	<0.01	36.2	N/A
	57F	–	Severe respiratory symptoms	–	–	MAHA, thrombocytopenia	7.5	7.9	98	1150	–	–	–	–	Schistocytes	0.86%	25.3	N/A
	38 M	–	Rectorrhagia for 1 week	–	–	MAHA, thrombocytopenia	13.5	8	5	545	–	–	–	–	Schistocytes	0.06	14	N/A
Shankar et al. [10]	30 M	None	Low back pain, left flank pain and hematuria	No	7 days	MAHA, thrombocytopenia, kidney dysfunction	10	13.7	9	1068	1.51	<0.1	2.8	–	–	3%	0.6	7
Nicolotti et al. [11]	44F	Obesity, DVT	Weakness, dizziness, abdominal discomfort	Yes	4 days	MAHA, thrombocytopenia, neurological dysfunction, kidney injury	–	6.7	7	2961	2.3	<0.1	–	2	Schistocytes	<5%	57	5

Abbreviations: CHF, congestive heart failure; Diff, differential; DVT, deep vein thrombosis; HLD, hyperlipidemia; HTN, hypertension; IVIG, intravenous immunoglobulin; LDH, lactate dehydrogenase; MAHA, microangiopathic hemolytic anemia; MI, myocardial infarction; PLT, platelet; SOB, shortness of breath; TTP, thrombotic thrombocytopenic purpura; WBC, white blood cell.

Interestingly, in a cross‐sectional study by Mancini et al. of 50 patients to investigate the mechanism of microthrombosis in COVID‐19 progression, more significant alterations of the vWF‐ADAMTS13 axis were associated with increasing severity of COVID‐19 infection. Patients requiring higher intensity care were found to have a higher vWF:Ag to ADAMTS‐AL ratio, and therefore likely more hypercoagulable and pro‐thrombotic [16]. vWF:Ag was not obtained in our hospital nor mentioned in most reported cases. We propose collecting this information in those with suspected TTP in order to better characterize the underlying pathophysiology. Surprisingly, none of these subjects exhibited thrombocytopenia suggesting the absolute level of vWF:Ag alone does not lead to TTP. Interestingly, a moderate ADAMTS‐AL reduction was noted in severe infection. As supported by Maharaj et al., these findings suggest that severe COVID‐19 infection may alter other pathways that interact with the classical vWF‐ADAMTS13‐axis via a currently undescribed mechanism, leading to a prothrombotic state [17].

In our case series and review, although it is difficult to surmise from such a small number of cases, several findings suggest a confluence of insults may be uniquely involved in aTTP after COVID‐19 infection. The limitations to our study include the size of our series, namely only three patients are included, and 12 additional case reports were found in the literature, and there are other confounding diseases and medical history for the 15 participants that could predispose them to TTP. Nonetheless, we surmise that there are two types of disease pathogenesis. Firstly, those with a prior COVID‐19 infection, that is, who were IgG positive—we propose that the primary pathology is molecular mimcry predisposed them to TTP. Secondly, those with an acute COVID‐19 infection have an acutly dysregulated vWF: ADAMTS‐13 pathway, as described in Mancini et al. and Maharaj et al., which in certain cases can progress to TTP. The three patients who were only COVID IgG‐positive, meaning they had a COVID infection previously, had medical histories including breast cancer in remission, infection with cholangitis, and prior TTP from bacterial pneumonia. Our first patient had a family history of alpha‐thalassemia trait and a family history of autoimmune conditions including ITP, and we propose that ones genetics can predispose one to other autoimmune conditions. Additionally, this patient had cholangitis, and there has been a case report that describes cholangiopancreatography as a rare cause of TTP [18]. In this case, an association with COVID‐19 is in fact difficult to demonstrate since he had no symptoms of COVID‐19; therefore a close association with COVID‐19 is possible but cannot be confirmed. Breast cancer has been associated with thrombotic microangiopathies leading to thrombocytopenia, and we propose that our second patient's breast cancer history predisposed her to TTP. For our third patient, whose outcome was fatal, a prior TTP incident predisposes one to a second incident [19]. Additionally, there may be another type of microangiopathy with sepsis that further complicated his hospital course and fatal outcome. We propose that an autoimmune process was the tipping factor. Contrarily, for those patients with acute COVID‐19 infections two patients were pregnant, two had prior breast cancer in remission, one had hypothyroidism, and one was prescribed immunosuppression for a transplanted kidney. For those with an acute COVID‐19 infection causing significant alterations of the vWF‐ADAMTS13 axis (as described by Mancini et al.) as well as increased vWF, which may have lowered the amount of circulating ADAMTS‐13, made these patients more susceptible to aTTP. In either the prior or acute COVID‐19 infection, our observations suggest that these patients had a “perfect storm,” multiple factors causing insult to the ADAMTS‐13 pathway tipping them into TTP.

Several treatments are available for aTTP, which include PLEX, steroids, caplacizumab, and rituximab. PLEX is the staple for treatment of this life‐threatening, hematologic emergency. PLEX is continued daily until platelets recover. In addition, in 2020 the International Society on Thrombosis and Haemostasis recommended adding steroids—prednisone for less severe disease and high dose methylprednisolone for more severe disease [20]. Rituximab is normally used once severe disease is confirmed through ADAMTS‐13 levels. Caplacizumab is a nanobody that binds the A1 domain of vWF, blocking its interaction with the glycoprotein (GP) Ib‐IX‐V complex platelet‐receptor and therefore preventing platelet aggregation [21]. These treatments appear to remain very effective for aTTP associated with COVID‐19 infection.

## 3 CONCLUSION

This abstract uniquely describes a case series of aTTP triggered by COVID‐19. We found two sets of patients, those with an acute COVID‐19 infection and those with a prior COVID‐19 infection. Likely molecular mimicry plays a role in both forms of aTTP. In acute illness, reduced levels of ADAMTS‐13 may progress into aTTP. For those with a prior COVID‐19 infection, molecular mimicry is likely the predominant trigger, but having an underlying predisposition is needed. Further investigation into this association and its possible mechanisms and a more detailed investigation into the genetics of those who acquire TTP after COVID‐19 infections are proposed. Also, obtaining levels of vWF:Ag may also be warranted for COVID‐19‐induced TTP. Understanding these mechanisms and identifying patients at hospital presentation through calculating PLASMIC scores are important for early treatment of this deadly disease.

## AUTHOR CONTRIBUTIONS

Melissa Neumann is main author, researcher, writer, and editor of manuscript. Adit Singhal completed table/chart, is researcher, writer, and editor. Junaid Habibullah edited and provided high‐level guidance and feedback. Sean Dhar edited and provided high‐level strategic insights and guidance.

## CONFLICT OF INTEREST

The authors declare they have no conflicts of interest.

## FUNDING INFORMATION

The authors received no specific funding for this work.

## ETHICS STATEMENT

Ethical approval is not required for this study in accordance with local or national guidelines. Written informed consent was obtained from the patients for publication of the details of their medical case and any accompanying images.

## Data Availability

All data generated or analyzed during this study are included in this article. Further enquiries can be directed to the corresponding author.
